# Promoter DNA methylation regulates progranulin expression and is altered in FTLD

**DOI:** 10.1186/2051-5960-1-16

**Published:** 2013-05-13

**Authors:** Julia Banzhaf-Strathmann, Rainer Claus, Oliver Mücke, Kristin Rentzsch, Julie van der Zee, Sebastiaan Engelborghs, Peter P De Deyn, Marc Cruts, Christine van Broeckhoven, Christoph Plass, Dieter Edbauer

**Affiliations:** 1 German Center for Neurodegenerative Diseases (DZNE), University of Munich, Schillerstr. 44, Munich 80336, Germany; 2 Division Epigenomics and Cancer Risk Factors, German Cancer Research Center, Im Neuenheimer Feld 280, Heidelberg 69120, Germany; 3 Department of Hematology/Oncology, University Medical Center Freiburg, Hugstetterstr. 55, Freiburg 79106, Germany; 4 VIB Department of Molecular Genetics, Neurodegenerative Brain Diseases Group, Universiteitsplein 1, Antwerp, Belgium; 5 Institute Born-Bunge, University of Antwerp, Universiteitsplein 1, Antwerp, Belgium; 6 Department of Neurology and Memory Clinic, Antwerp Hospital Network Middelheim and Hoge Beuken, Antwerp, Belgium; 7 Adolf-Butenandt-Institute, Biochemistry, Ludwig-Maximilians-University, Schillerstr. 44, Munich 80336, Germany

**Keywords:** 5-aza-2^′^-deoxycytidine, DNA methylation, Epigenetics, FTLD, Progranulin

## Abstract

**Background:**

Frontotemporal lobar degeneration (FTLD) is a heterogeneous group of neurodegenerative diseases associated with personality changes and progressive dementia. Loss-of-function mutations in the growth factor progranulin (GRN) cause autosomal dominant FTLD, but so far the pathomechanism of sporadic FTLD is unclear.

**Results:**

We analyzed whether DNA methylation in the GRN core promoter restricts GRN expression and, thus, might promote FTLD in the absence of GRN mutations. GRN expression in human lymphoblast cell lines is negatively correlated with methylation at several CpG units within the GRN promoter. Chronic treatment with the DNA methyltransferase inhibitor 5-aza-2^′^-deoxycytidine (DAC) strongly induces GRN mRNA and protein levels. In a reporter assay, CpG methylation blocks transcriptional activity of the GRN core promoter. In brains of FTLD patients several CpG units in the GRN promoter are significantly hypermethylated compared to age-matched healthy controls, Alzheimer and Parkinson patients. These CpG motifs are critical for GRN promoter activity in reporter assays. Furthermore, DNA methyltransferase 3a (DNMT3a) is upregulated in FTLD patients and overexpression of DNMT3a reduces GRN promoter activity and expression.

**Conclusion:**

These data suggest that altered DNA methylation is a novel pathomechanism for FTLD that is potentially amenable to targeted pharmacotherapy.

## Background

Frontotemporal Lobar Degeneration (FTLD) is the second most common cause of presenile dementia in patients under 60 years of age and accounts for approximately 5 to 10% of all patients suffering from dementia [1]. In FTLD, the progressive neurodegeneration in the frontal and temporal lobes is accompanied by proteinaceous intraneuronal inclusions (reviewed in [2]), which allow for pathological stratification into FTLD-subtypes: FTLD-tau and FTLD-FUS are characterized by inclusions of microtubule associated protein tau (MAPT) and fused in sarcoma (FUS), respectively, whereas inclusions found in FTLD-TDP stain positive for ubiquitin and TAR DNA binding protein 43 (TDP-43) [3,4]. While the majority of FTLD-cases occurs sporadically, 10 to 40% of FTLD patients have a positive family history with hexanucleotide repeat expansions in the uncharacterized gene C9ORF72 [5-7] or with mutations in the genes coding for TDP-43 (TARDBP), valosin-containing protein (VCP) [8,9], or in the growth factor progranulin (GRN) [10,11].

Approximately 70 FTLD-TDP-associated autosomal dominant mutations in the GRN gene are known (http://www.molgen.vib-ua.be/FTDMutations/). Pathogenic mutations inhibit expression, secretion or activity of GRN from one allele, resulting in haploinsufficiency [12]. In neurons, GRN acts as a neurotrophic factor and is crucial for proper morphology and connectivity [13]. Reduced GRN levels presumably fail to sustain proper function and survival in aged neurons and, thus, eventually lead to progressive neurodegeneration in FTLD [14-16].

Despite their germline mutation in the GRN gene, mutation carriers for unclear reasons often show strikingly asymmetric cortical atrophy, which does not usually occur in FTLD-tau or FTLD-TDP cases with C9ORF72 hexanucleotide repeat expansions [17,18]. It is tempting to speculate that environmental factors and subsequent epigenetic changes might contribute to unilateral disease progression, as environmental factors have been shown to modulate DNA methylation patterns in humans [19]. Histone deacetylase inhibitors boost GRN expression levels [20], however, little is known about the (patho)physiological mechanisms that govern GRN expression.

Next to histone modifications, DNA methylation is the most widely studied mechanism of epigenetic gene regulation. DNA hypermethylation at the 5′-position of cytosine nucleotides that are followed by guanine nucleotides (CpG dinucleotides) is associated with gene silencing when occurring in normally unmethylated CpG-rich promoter regions [21].

We tested the hypothesis that aberrant DNA methylation in the promoter region of GRN might contribute to the pathogenesis of FTLD. Here, we show that GRN expression is regulated by DNA methylation at several CpG units in its promoter region, and we found that GRN methylation is altered in FTLD patients compared to healthy controls, possibly through altered expression of DNA methyltransferases.

## Results

### GRN expression is inversely correlated to promoter methylation

**Figure 1:**
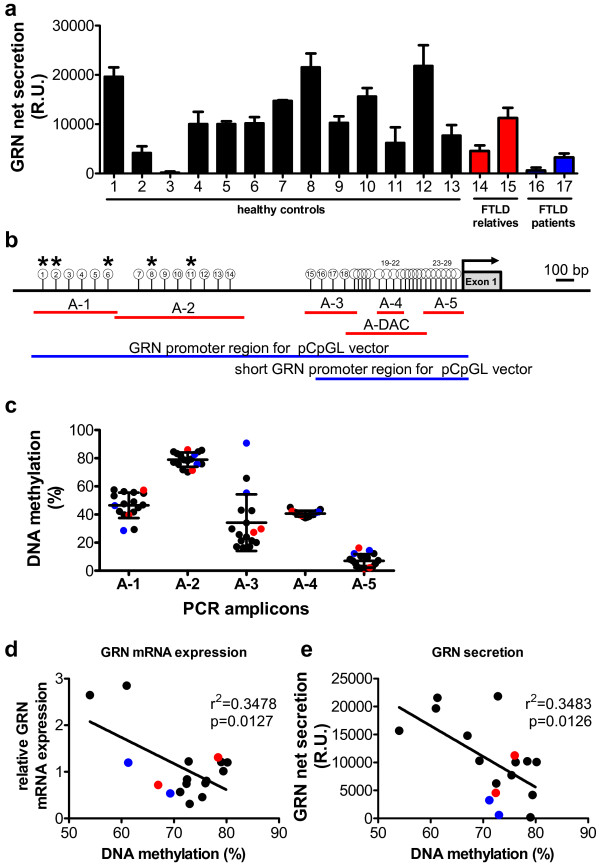
**GRN expression in human lymphoblast cell lines is inversely correlated to its promoter methylation.** (**a**) GRN net secretion was measured by ELISA in LCLs derived from neurologically healthy individuals (LCL #1-13), unaffected relatives of FTLD patients (LCL #14, 15, highlighted in blue) and FTLD-patients (LCL#16, 17, highlighted in red). n = 3, mean ± SEM. (**b**) Scheme of GRN promoter region. Red bars depict PCR-amplicons analyzed for DNA methylation levels by MassARRAY. Blue bars indicate full length and short promoter region that was cloned into the pCpGL vector for luciferase assays (compare Figure 3). White circles display CpG units in amplicons A-1 to A-5 and A-DAC quantified by MassARRAY. CpG units that were not analyzed are not shown. Asterisks indicate significant correlation between GRN mRNA expression or GRN secretion and GRN methylation at respective CpG unit (*p < 0.05, linear regression analysis, Benjamini Hochberg multiple testing and FDR correction, compare Additional file 1: Table S3). (**c**) Average DNA methylation levels in amplicons A-1 to A-5 for individual LCLs are plotted. Mean ± SD. Color code as in (a). (**d**) Correlation between GRN mRNA expression and average DNA methylation at CpG units 1, 2, 6, 8 and 11 is shown. GRN mRNA expression was quantified by qPCR and normalized to PGK1 and GAPDH. Relative mRNA expression levels were plotted against average DNA methylation levels. Correlation between parameters was quantified by linear regression analysis, r^2^ and p-values are given. Color code as in (a). (**e**) Correlation between GRN secretion and average DNA methylation at CpG units 1, 2, 6, 8 and 11. GRN secretion was determined by ELISA and relative units (R.U.) were plotted against average DNA methylation levels. Correlation between parameters was quantified by linear regression analysis, r^2^ and p-values are given. Color code as in (a).

To address whether GRN expression is regulated by epigenetic mechanisms we analyzed the net level of GRN secretion, which is composed of cellular GRN secretion, re-uptake and degradation (called net secretion in the following) in lymphoblast cell lines from 13 healthy individuals, two FTLD patients’ relatives, both carrying the genetic variant V514M in the GRN gene (LCLs #14, 15), and two FTLD patients with the genetic variants R298H (LCL#16) and R432C (LCL #17) in the GRN gene. 24 h after seeding identical cell numbers, the amount of net secreted GRN varied significantly with over 100-fold difference between the lowest and highest expressing cell line (LCL #3 vs. LCL #12 (Figure 1a)). We hypothesize that this large variation in GRN net secretion among different LCLs is partly due to differences in DNA methylation in the GRN promoter and other clonal effects.

To test if DNA methylation plays a role in the regulation of GRN expression, genomic DNA from LCLs was subjected to bisulfite treatment that specifically converts all un-methylated cytosines to uracils, whereas methylated cytosines remain unaffected. We subsequently amplified five amplicons (A-1 to A-5) by PCR, which were subjected to quantitative DNA methylation analysis using the MassARRAY platform as previously described [22] (Figure 1b). As the sequence context in the GRN promoter region is very GC rich, specific DNA amplification is difficult especially after bisulfite conversion. PCR amplification of other GRN promoter regions did not result in specific PCR products suitable for DNA methylation analysis. However, analysis of amplicons A-1 to A-5 allowed us to quantify the DNA methylation levels at 29 CpG units, consisting of one CpG dinucleotide and rarely of two CpG dinucleotides in case the two adjacent CpG sites were not separated by a specific RNase A cleavage site (pyrimidine nucleotides) (Additional file 1: Figure S1). Average DNA methylation levels varied in the different promoter/genomic regions ranging from 79% average DNA methylation in the distal A-2 region to 7% in A-5 directly upstream of the transcriptional start site (Figure 1c).

Next, we carried out linear regression analyses to elucidate whether GRN DNA methylation levels of the individual CpG units were inversely correlated to GRN mRNA expression and GRN net secretion levels. As depicted in Additional file 1: Table S3, we observed a significant negative correlation for mRNA expression and DNA methylation at CpG units 1, 6, 8, and 11. Moreover, CpG unit 2 was significantly negatively correlated to GRN net secretion (indicated with asterisks in Figure 1b). To illustrate the significant negative correlation, the mean of DNA methylation of these five significant CpG units (1, 2, 6, 8 and 11) was plotted against GRN mRNA expression (Figure 1d) and GRN net secretion levels (Figure 1e). Genetic variations that have been discussed to potentially alter GRN expression levels before (TMEM106b rs1990622 [23], GRN rs5848 [24] and SORT1 rs646776 [25]), did neither correlate with different GRN DNA methylation levels nor GRN mRNA/protein expression (Additional file 1: Figure S2).

From these results we conclude that methylation levels of specific CpG units in the GRN promoter 1–2 kb distal of the transcriptional start site regulate GRN mRNA expression and protein secretion in human cell lines.

### 5-aza-2′-deoxycytidine (DAC) treatment leads to re-expression of GRN by reducing GRN promoter methylation

**Figure 2:**
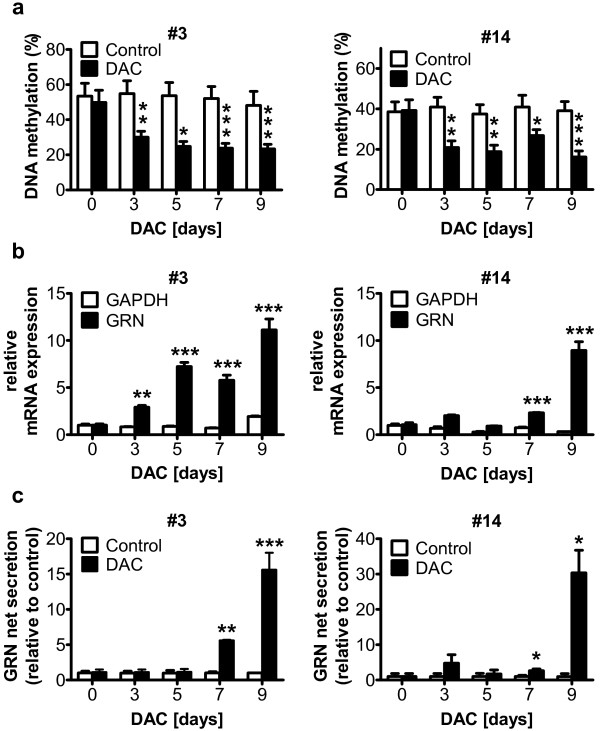
**The DNMT-inhibitor 5-aza-2′-deoxycytidine (DAC) reduces DNA methylation in the GRN promoter and increases GRN expression in LCLs.** LCL #3 and #14 were treated daily with 0.5 μM DAC for 9 days. (**a**) Quantitative DNA methylation analysis of amplicon A-DAC (compare Figure 1b) shows progressive demethylation in the GRN promoter region. Average DNA methylation across all 15 CpG units in the A-DAC amplicon is shown. Mean ± SD, *p < 0.05, **p < 0.01, ***p < 0.001, Student’s t-test. (**b**) GRN mRNA expression levels were analyzed by qPCR and normalized to PGK1. GRN mRNA expression strongly increased, while the expression of GAPDH did not change. Mean ± SD, n = 3, **p < 0.01, ***p < 0.001, ANOVA with Tukey’s Multiple Comparison test. (**c**) GRN secretion was quantified by ELISA and normalized to absolute cell number. Chronic treatment of LCL #3 and LCL #14 with 0.5 μM DAC for 9 days significantly increased GRN protein secretion into the cell culture supernatant, while the secretion in mock treated control cells remained low. Mean ± SD, n = 3, **p < 0.01, ***p < 0.001, ANOVA with Tukey’s Multiple Comparison test.

Next, we asked whether inhibition of DNA methyltransferases (DNMTs) would lead to re-expression of GRN mRNA by reducing DNA hypermethylation. We treated LCL #3 and LCL #14 with 0.5 μM of the DNMT inhibitor 5-aza-2′-deoxycytidine (DAC) for 9 days, which leads to progressive DNA demethylation upon DNA replication in proliferating cells [26]. We analyzed DNA methylation in one large amplicon (A-DAC) in the GRN promoter region by MassARRAY (Figure 1b and Additional file 1: Figure S1), covering 15 CpG units. As expected, in LCL #3 the average DNA methylation level within A-DAC was significantly reduced to 22% upon prolonged DAC treatment compared to a stable methylation rate of 55% in untreated samples. In LCL #14 DAC treatment also caused a significant reduction in GRN promoter methylation down to 16% after 9 days of treatment, while untreated cells showed 40% DNA methylation (Figure 2a).

To test whether reduced DNA methylation enhances GRN transcription, we analyzed GRN mRNA expression in cells treated with DAC by qPCR. As shown in Figure 2b, treatment with 0.5 μM DAC over 9 days led to a progressive highly significant 11-fold increase in GRN mRNA expression in LCL #3. Similarly, LCL #14 also showed highly significant 9-fold GRN mRNA induction after 9 days of DAC treatment, while the mRNA expression of the commonly used housekeeping gene GAPDH remained stable over time in both cell lines.

In parallel to GRN mRNA expression levels, GRN net secretion in LCL #3 significantly increased 7-fold and 15-fold after 7 and 9 days of DAC treatment, respectively (Figure 2c). In LCL #14, DAC treatment even led to a 30-fold increase of GRN secretion after 9 days, while GRN-secretion in control cells remained stable over time (Figure 2c).

Enhanced GRN expression upon DNA demethylation through chronic DAC treatment suggests that endogenous methylation in the GRN promoter restricts GRN expression.

### GRN promoter activity is regulated by DNA methylation at distinct CpG units

**Figure 3:**
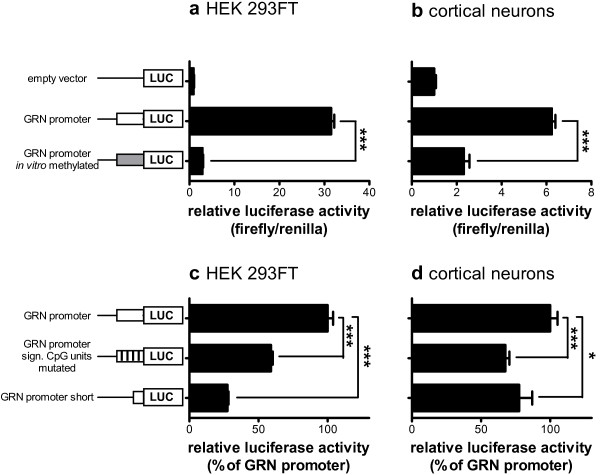
**DNA methylation inhibits GRN promoter activity at distinct CpG units.***In vitro* methylated and unmethylated pCpGL plasmids containing the GRN core promoter region driving expression of firefly luciferase were transiently cotransfected into (**a**) HEK 293FT cells and (**b**) primary rat cortical neurons together with a *Renilla* luciferase expressing plasmid. The full length GRN promoter pCpGL plasmid, a GRN promoter construct with site specific mutations of the significant CpG units in amplicons A-1 and A-2, and a short GRN promoter construct lacking amplicons A-1 and A-2 were transiently cotransfected into (**c**) HEK 293FT cells and (**d**) primary rat cortical neurons together with a *Renilla* luciferase expressing plasmid. Luciferase reporter activity was measured 48 h (a + c, HEK 293FT) or 72 h (b + d, neurons) after transfection. Relative luciferase activity was determined by normalizing firefly luciferase against *Renilla* luciferase activity. The empty vector was used as negative control. Mean ± SEM, n ≥ 3. ***p < 0.001, Student’s t-test, *sign*. significant.

To directly analyze the effect of GRN promoter methylation on mRNA expression we used luciferase reporter assays. The GRN promoter region (from −2423 to +207 bp relative to the transcriptional start site (Figure 1b)) was cloned upstream of firefly luciferase into pCpGL, a vector completely devoid of CpG motifs [27]. The putative GRN promoter region enhanced luciferase activity 32-fold compared to the empty vector upon transient transfection of HEK 293FT cells. *In vitro* methylation using excess enzyme to guarantee complete methylation of the reporter plasmid prior to transfection, reduced GRN promoter activity almost to background levels (Figure 3a). This further supports our hypothesis that promoter methylation regulates GRN expression. Since FTLD mainly manifests in the cerebral cortex, we repeated these experiments in rat cortical neurons transfected after five days in culture with the methylated and unmethylated GRN promoter constructs. In accordance to our findings in HEK 293FT cells, the GRN promoter activity in primary neurons was almost completely blocked by *in vitro* methylation in the luciferase reporter assay (Figure 3b).

Next, we tested whether the five CpG units 1, 2, 6, 8 and 11 that significantly correlated with reduced GRN expression (see Figure 1) are crucial for maintaining full GRN promoter activity. Because CpG methylation is thought to block binding of transcription factors, we mutated those five CpG dinucleotides to TpG dinucleotides, which may also block binding of such transcription factors. In addition, we cloned a short GRN promoter construct which is devoid of amplicons A-1 and A-2, in which the significant CpG units are located. Transient transfection of the mutated GRN promoter construct into HEK 293FT cells significantly reduced GRN promoter activity by more than 40% compared to the wildtype GRN promoter construct. Transfection of the truncated GRN promoter led to a highly significantly reduced promoter activity of more than 70% (Figure 3c). We confirmed these findings by transfecting rat primary cortical neurons with the mutated and short GRN promoter constructs, which reduced the GRN promoter activity by more than 30% and 20%, respectively, compared to the wildtype GRN promoter construct (Figure 3d).

In summary, our *in vitro* data demonstrate that CpG methylation in the GRN core promoter region strongly inhibits its transcriptional activity. Further, the presence of the CpG units that showed a significant inverse correlation between DNA methylation and GRN expression are crucial for maintaining full GRN promoter activity.

### The GRN promoter region is hypermethylated in brains from FTLD patients

**Figure 4:**
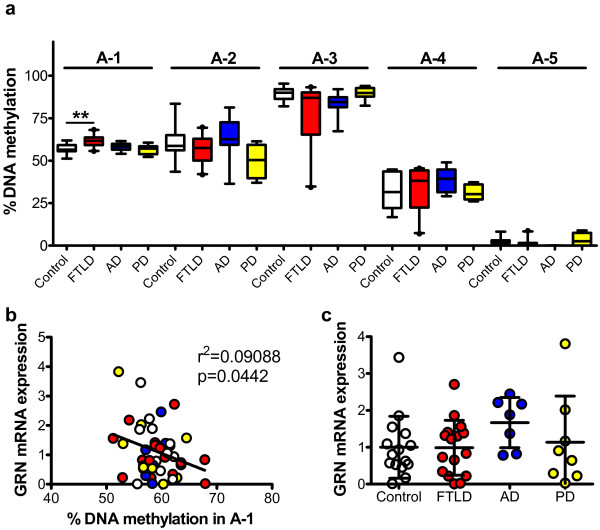
**GRN promoter DNA methylation is altered in FTLD-TDP patients.** Genomic DNA from human frontal cortex samples was subjected to bisulfite conversion to analyze DNA methylation in the GRN promoter by MassARRAY (See Figure 1b). (**a**) Box plots show average percentage of DNA methylation in amplicons A-1 to A-5 comparing healthy controls (white), FTLD patients (red), AD patients (blue) and PD patients (yellow). Whiskers depict 5–95 percentile, **p < 0.01, Kruskal Wallis test with Dunn’s Multiple Comparison Test. (**b**) Negative correlation of average DNA methylation across amplicon A-1 to GRN mRNA expression. Correlation between parameters was quantified by linear regression analysis, r^2^ and p-values are given. Color code as in (**a**). (**c**) Relative GRN mRNA expression levels in brains from controls, FTLD, AD and PD patients quantified by qPCR and normalized to the housekeeping genes PGK1 and GAPDH. Mean ± SD.

After having demonstrated that DNA methylation regulates GRN promoter activity, we asked whether patients who suffered from FTLD show aberrant GRN promoter methylation in the brain. Therefore, we analyzed DNA methylation in the frontal cortices of 20 FTLD patients (pathological diagnosis FTLD-TDP [28,29], average age at disease onset 60 ± 10.2 years, average age at death 66 ± 10.6 years), 8 AD patients (average age at onset: 69 ± 14.8 years, age at death: 80 ± 10.6 years), 8 PD patients (average age at onset: 67 ± 16.2 years, age at death: 79 ± 8.4 years) and 15 healthy, age-matched controls (average age at death 67 ± 10.1 years) (Table 1). We isolated genomic DNA from human brain samples, performed bisulfite conversion, amplified five regions (A-1 through A-5) upstream of the transcriptional start site of GRN (compare Figure 1b), and subjected the PCR products to quantitative methylation analysis. Similar to LCLs, the average DNA methylation levels varied in the different amplicons analyzed: Highest (and most variable) average DNA methylation was observed in A3 (average 85%), whereas CpG sites in the amplicon A-5 located directly upstream of the transcriptional start site was much less methylated (average 2%, Figure 4a).

**Table 1:** Pathological, clinical and genetic info of human brain samples used

		**Clinical info**							**Genetic info**				
**Origin**	Pathological diagnosis*^1^	**Clinical diagnosis**	**Sub-class**	**Gender**	**Age at onset**	**Age at death**	**Fam/Spor**	**Pathogenic Mutation**	**TMEM106B rs1990622^2^**	**GRN rs5848^3^**	**C9ORF72**	**SORT1^4^**	**RNA quality ok**
VIB	Def. Control	N.A.	N.A.	m	N.A.	78.1	N.A.	N.A.	CT	CC	no	AG	yes
VIB	Def. Control	N.A.	N.A.	m	N.A.	66.3	N.A.	N.A.	CT	CC	no	AA	yes
VIB	Def. Control	N.A.	N.A.	m	N.A.	73.3	N.A.	N.A.	TT	TC	no	AA	yes
VIB	Def. Control	N.A.	N.A.	f	N.A.	62.7	N.A.	N.A.	TT	TC	no	AA	yes
VIB	Def. Control	N.A.	N.A.	m	N.A.	64.6	N.A.	N.A.	CT	TT	no	AG	yes
MRC	Def. Control	N.A.	N.A.	m	N.A.	77	N.A.	N.A.	CC	TC	no	AG	yes
MRC	Def. Control	N.A.	N.A.	m	N.A.	66	N.A.	N.A.	CT	CC	no	AG	yes
MRC	Def. Control	N.A.	N.A.	m	N.A.	54	N.A.	N.A.	TT	CC	no	AA	yes
MRC	Def. Control	N.A.	N.A.	m	N.A.	59	N.A.	N.A.	TT	TC	no	AG	yes
MRC	Def. Control	N.A.	N.A.	m	N.A.	55	N.A.	N.A.	CC	CC	no	AA	yes
MRC	Def. Control	N.A.	N.A.	m	N.A.	67	N.A.	N.A.	CT	TC	no	AA	yes
MRC	Def. Control	N.A.	N.A.	m	N.A.	78	N.A.	N.A.	CT	CC	no	AA	yes
MRC	Def. Control	N.A.	N.A.	m	N.A.	79	N.A.	N.A.	TT	TC	no	AG	yes
MRC	Def. Control	N.A.	N.A.	m	N.A.	50	N.A.	N.A.	TT	CC	no	AA	yes
MRC	Def. Control	N.A.	N.A.	m	N.A.	82	N.A.	N.A.	CT	CC	no	AG	yes
VIB	FTLD-TDP	MXD	N.A.	m	72	83	S	no	CT	CC	no	AA	yes
VIB	FTLD-TDP B	FTLD	FTD	m	47	50	F	no	CT	CC	no	AG	yes
VIB	FTLD-TDP	FTLD	prob AD	f	80	88	F	no	CT	TT	no	AA	yes
VIB	FTLD-TDP B	FTLD-ALS	FTD-ALS	m	59	62	S	no	CT	CT	no	AG	yes
VIB	FTLD-TDP D	FTLD	FTD	f	44	56	F-AD	VCP Arg159His^5^	TT	TC	no	AA	yes
VIB	FTLD-TDP D	FTLD	FTD	m	63	68	F-AD	VCP Arg159His^5^	CT	TT	no	AA	yes
VIB	FTLD-TDP A	FTLD	N.A.	f	62	68	F-AD	GRN IVS1 + 5G > C^6^	CT	TC	no	AA	yes
VIB	FTLD-TDP A	FTLD	N.A.	f	58	63	F-AD	GRN IVS1 + 5G > C^6^	TT	TC	no	AG	yes
VIB	FTLD-TDP A	FTLD	N.A.	m	57	62	F-AD	GRN IVS1 + 5G > C^6^	CT	TC	no	AG	yes
VIB	FTLD-TDP A	FTLD	FTD	f	69	75	F-AD	GRN IVS1 + 5G > C^6^	TT	TC	no	AA	yes
MRC	FTLD-TDP B	FTLD	FTD + MND	m	43	45	S	no	CT	CC	no	AG	yes
MRC	FTLD-TDP B	FTLD	FTD + MND	m	65	67	S	no	CC	CC	no	AG	yes
MRC	FTLD-TDP B	FTLD	FTD + MND	m	74	76	S	no	TT	TC	no	AG	no
MRC	FTLD-TDP B	FTLD	FTD	m	60	68	S	no	TT	CC	no	AA	yes
MRC	FTLD-TDP B	FTLD	FTD	m	45	51	S	no	CT	TT	no	AG	yes
MRC	FTLD-TDP B	FTLD	FTD	m	59	66	S	no	TT	CC	no	AA	yes
MRC	FTLD-TDP B	FTLD	FTD + MND	m	58	69	S	no	CC	CC	no	AA	yes
MRC	FTLD-TDP B	FTLD	FTD	m	58	66	S	no	CT	CC	no	AG	no
MRC	FTLD-TDP B	FTLD	FTD/SD	m	68	74	S	no	TT	CC	no	AA	yes
MRC	FTLD-TDP B	FTLD	MND	m	N.A.	71	S	N.A.	TT	TC	no	AA	no
VIB	AD-CAA	Prob AD		f	61	75	F	APP -369C/G^7^	CT	CC	no	AG	yes
VIB	AD	Prob AD		f	-	85	S	no	CT	TC	no	AA	no
VIB	AD	Prob AD		m	80	86	S	no	TT	TC	no	AG	yes
VIB	AD	Prob AD		m	87	91	U	no	CC	CC	no	AG	yes
VIB	AD	Prob AD		m	67	77	F	no	TT	TC	no	AG	yes
VIB	AD	Prob AD		f	64	79	S	no	CT	TT	no	AA	no
VIB	AD	Poss AD		m	<84	87	S	no	CT	CC	no	AA	yes
VIB	AD	Prob AD		f	50	57	F	PSEN1 P264L^8^	TT	TC	no	AA	yes
MRC	PD	PD		f	45	62	N.A.	N.A.	CC	CC	no	AA	yes
MRC	PD	Dementia		f	~84	89	N.A.	N.A.	CT	TC	no	AA	yes
MRC	PD	PD		f	~80	85	N.A.	N.A.	CC	CC	no	AG	yes
MRC	PD	PD		m	?	73	N.A.	N.A.	CC	CC	no	AG	yes
MRC	PD	?		m	?	76	N.A.	N.A.	CC	CC	no	AA	yes
MRC	PD	PD		f	76	80	N.A.	N.A.	CT	TC	no	AG	yes
MRC	PD	PD		m	66	79	N.A.	N.A.	CT	TC	no	AA	yes
MRC	PD	PD?/AD		m	82	84	N.A.	N.A.	CT	TC	no	AA	yes

* according to current classification criteria as proposed in [28],

^1^[29],

^2^[30],

^3^[31],

^4^(van der Zee J. et al., unpublished data),

^5^[32],

^6^[10],

^7^[33],

^8^[34]

*AD* Alzheimer’s disease, *ALS* amyothropic lateral sclerosis, *APP* amyloid precursor protein, *CAA* Cerebral Amyloid Angiopathy, *F* familial, *F*-*AD* familial autosomal dominant, *IVS* splice donor site of intron 1 *MND* motorneuron disease, *MRC* Medical Research Council London Neurodegenerative Diseases Brain Bank, *MXD* mixed dementia, *N*.*A*. not applicable, *PD* Parkinson’s disease, *Prob* probably, *Poss* possibly, *S* sporadic, *SD* semantic dementia, *PSEN1* presenilin 1, *U* unknown, *VIB* VIB Department of Molecular Genetics, Antwerp, Belgium.

By comparing average DNA methylation levels between all FTLD patients, AD patients, PD patients and controls across the different amplicons, we observed a slight, but highly significant increase in DNA methylation from 57% to 62% in FTLD patients in amplicon A-1. DNA methylation levels in amplicons A-2 to A-5 did not differ significantly between FTLD, AD, PD patients and healthy controls (Figure 4a).

As we had observed an inverse correlation between GRN promoter methylation levels and GRN mRNA expression and net secretion in LCLs (Figure 1d + e), we next analyzed GRN mRNA expression levels in patient and control brain by qPCR. Elevated DNA methylation levels in amplicon A-1 were significantly correlated to reduced GRN mRNA expression as depicted in Figure 4b, indicating that epigenetic mechanisms may contribute to the regulation of GRN expression *in vivo*. However, we did not observe a significant difference in overall GRN mRNA expression levels between FTLD patients and AD or PD patients or healthy controls (Figure 4c). Furthermore, SNPs in TMEM106b (rs1990622), GRN (rs5848) and SORT1 (rs646776) did not correlate with different GRN DNA methylation or GRN expression levels (Additional file 1: Figure S3).

In summary, FTLD patients show altered GRN promoter methylation suggesting that epigenetic mechanisms may play a role in the regulation of GRN expression *in vivo*. This finding is supported by a significant inverse correlation of GRN mRNA expression and DNA methylation levels in amplicon A-1 of the GRN promoter in human brain samples.

### DNMT levels are altered in FTLD patients

**Figure 5:**
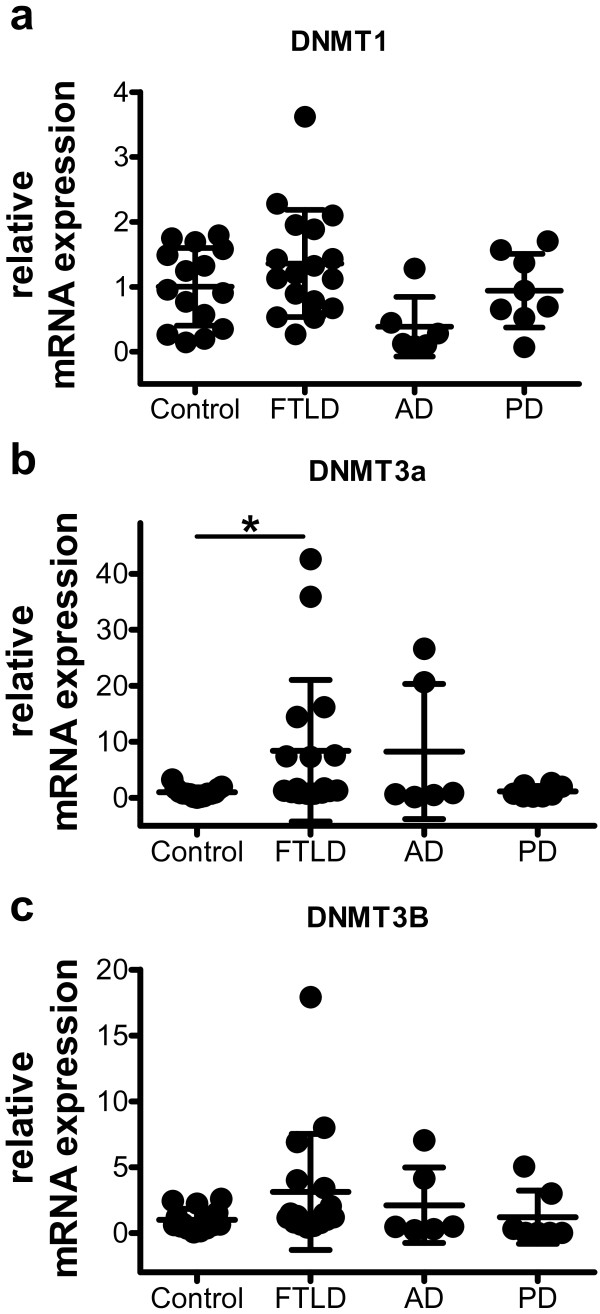
**In FTLD-TDP patients, DNMT3a mRNA expression levels are altered.** Relative mRNA expression levels of DNMT1 (**a**), DNMT3a (**b**) and DNMT3b (**c**) in brain samples quantified by qPCR and normalized to the housekeeping genes PGK1 and GAPDH. Mean ± SD, *p < 0.05, Kruskal Wallis test with Dunn’s Multiple Comparison Test.

Cellular DNA methylation is orchestrated by three distinct DNA methyltransferases, DNMT1, DNMT3a and DNMT3b, and aberrant expression of these DNMTs has been causally linked to atypical DNA methylation levels in human cancers [35]. Therefore, we analyzed DNMT mRNA expression levels in the brain samples from FTLD patients and healthy controls. While the expression levels of DNMT1 were unaffected (Figure 5a), DNMT3a levels were significantly increased 8.4-fold in FTLD patients compared to healthy controls (Figure 5b). DNMT3b levels showed a trend towards increased expression, not reaching statistical significance (Figure 5c). Therefore, we conclude that elevated DNMT3a expression may contribute to the aberrant DNA methylation levels found in FTLD patients.

### Overexpression of DNMT3a alters GRN promoter activity in primary cortical neurons and reduces GRN mRNA expression in LCLs

**Figure 6:**
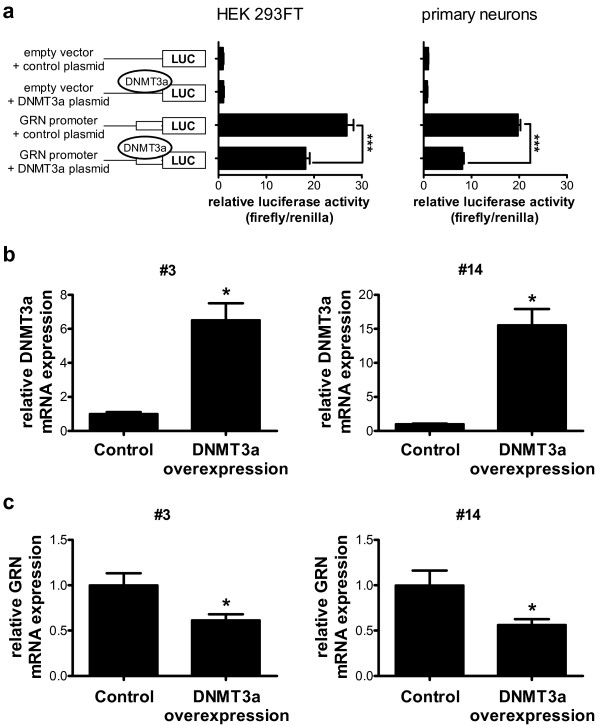
**Overexpression of DNMT3a alters GRN promoter activity in primary cortical neurons and reduces GRN mRNA expression in LCLs.** (**a**) pCpGL plasmid containing the GRN core promoter and a DNMT3a overexpression construct were transiently transfected in HEK 293FT cells (left panel) and in rat primary cortical neurons (right panel). Relative luciferase activity was determined by normalizing firefly luciferase against *Renilla* luciferase activity. Empty vectors were used as negative control. Firefly luciferase expression was significantly reduced upon DNMT3a overexpression. Mean ± SEM, n ≥ 3. ***p < 0.001, ANOVA with Tukey’s Multiple Comparison test. (**b**) Lentiviral expression of DNMT3a in LCLs #3 and #14. Overexpression was verified by qPCR five days after viral transduction. n = 5, mean ± SEM, *p < 0.05, Student’s t-test. (**c**) GRN mRNA expression levels were significantly reduced in DNMT3a overexpressing LCLs as quantified by qPCR and normalized to PGK1 expression levels. n = 5, mean ± SEM, *p < 0.05, Student’s t-test.

To elucidate whether altered DNMT expression levels can modify GRN promoter activity, we transfected HEK 293FT cells as well as primary cortical neurons with the pCpGL vector containing the GRN promoter region (compare Figure 1b) and a DNMT3a overexpression construct or the respective empty vector control. DNMT3a overexpression significantly reduced luciferase expression by more than 30% in HEK 293FT cells (Figure 1a) and by more than 50% in primary cortical neurons (Figure 6a), suggesting reduced GRN promoter activity upon methylation.

To further confirm that altered DNMT3a expression levels can modify GRN expression directly, we analyzed endogenous GRN mRNA expression after overexpression of DNMT3a in LCL#3 and #14 using lentiviral expression of DNMT3a. After five days of lentiviral transduction, DNMT3a levels were strongly elevated in both cell lines (Figure 6b). DNMT3a overexpression significantly reduced GRN mRNA expression by 71% in LCL#3 and by 44% in LCL#14 as quantified by qPCR (Figure 6c). In contrast, overexpression of DNMT1 did not alter GRN promoter activity and GRN mRNA expression (Additional file 1: Figure S4).

In summary, our data indicate that elevated DNMT3a levels in FTLD lead to reduction in GRN expression, presumably by enhancing DNA methylation levels in the GRN promoter, which results in reduced GRN promoter activity.

## Discussion

In the present study we demonstrate that GRN expression is regulated by DNA methylation in its core promoter region. FTLD patients show aberrant DNA methylation levels in the GRN promoter as well as altered expression of DNA methyltransferase 3a, indicating that epigenetic deregulation, particularly at the GRN promoter, may contribute to FTLD.

To study epigenetic alterations in FTLD we used lymphoblast cell lines (LCLs) derived from neurologically healthy subjects and FTLD patients. By correlating GRN mRNA expression and net secretion to DNA methylation levels in LCLs, we detected a significant inverse correlation in several CpG units in amplicon A-1 (Figure 1). Strikingly, DNA methylation levels in amplicon A-1 in brains of FTLD patients showed hypermethylation and an inverse correlation to GRN mRNA expression as well (Figure 4). This indicates that this region of the promoter might play an important role in epigenetic regulation of GRN expression. Historically, it was believed that DNA methylation exclusively in so-called CpG islands, regions with very high CpG density, is crucial for regulation of gene expression. However more recently, DNA methylation in regions with intermediate CpG density upstream of the CpG islands, so-called CpG island shores, has been strongly linked to gene expression [36]. The GRN promoter region is very CpG rich and it contains a CpG island covered by amplicons A-3 to A-5 according to prediction programs (e.g. http://dbcat.cgm.ntu.edu.tw/). Interestingly, the CpG units that show an inverse correlation with GRN expression are located within A-1 that display lower CpG density, representing a CpG island shore [36]. This strongly points towards epigenetic regulation of GRN expression in that particular region and supports our hypothesis that aberrant GRN methylation in amplicon A-1 regulates GRN expression *in vivo*.

Changes in DNA methylation levels have also been shown for amyotrophic lateral sclerosis (ALS) and AD. A genome-wide analysis of DNA methylation revealed several candidate genes that are aberrantly methylated in brain tissue of sporadic ALS patients that affect the expression of genes involved in calcium homeostasis, neurotransmission and oxidative stress [37]. In postmortem brain DNA analyses of AD patients, the DNA methylation status of telomerase reverse transcriptase (HTERT) was elevated compared to age-matched healthy individuals, however hypermethylation of HTERT was not accompanied by reduced mRNA expression in this study [38]. These findings are in accordance to our present data, as we detected a small, but highly significant increase in GRN promoter methylation, although we could not detect reduction in GRN mRNA expression. This discrepancy might have several experimental and biological reasons: First, genomic DNA itself and DNA methylation are far more stable than RNA [39] and thus post-mortem methylation levels more likely reflect the actual in vivo situation, highlighting the importance of epigenetic studies for biomarker discovery. Second, cellular heterogeneity may impair our analysis in the ground tissue from total frontal cortex, as we could not test whether aberrant GRN promoter methylation occurs only in a specific cell type or globally. Microglia activation is a common inflammatory response in FTLD brains (and other neurodegenerative diseases) [40]. Inflammation leads to increased microglial GRN expression [41], which may obscure a neuronal loss of GRN expression. A recent study demonstrated that FTLD patients with GRN haploinsufficiency surprisingly show elevated GRN mRNA expression levels in frontal cortex tissue despite reduced GRN serum levels [42]. Thus, inflammatory responses are likely to have dramatic effects on mRNA expression studies from the brain. Nevertheless, a recent publication supports our hypothesis that GRN expression is regulated by DNA methylation levels in FTLD. Galimberti and colleagues analyzed GRN promoter methylation at two CpG sites in close proximity to the transcriptional start site, as well as mRNA and protein expression levels in blood cells and plasma from FLTD patients [43]. Although they detected elevated methylation at these sites using a semi-quantitative PCR-based approach, they could not detect an inverse correlation between DNA methylation and GRN expression levels, suggesting that these CpG sites are not critical for regulating GRN expression. In contrast, we measured absolute quantities of DNA methylation across the 2500 nucleotide GRN core promoter region by automated mass spectrometry covering 29 CpG units in patient lymphoblast cells. With this in-depth analysis we found a significant inverse correlation for GRN expression and DNA methylation further upstream in the CpG island shore (amplicon A-1), which implies that this is the main regulatory region of GRN expression. In exactly this region we also detected elevated DNA methylation levels in brains from FTLD patients that were negatively correlated to GRN mRNA expression levels, suggesting that altered epigenetic marks may contribute to FTLD pathogenesis (Figure 4).

In order to identify the mechanism of how altered DNA methylation levels in the promoter region of GRN are established, we analyzed mRNA expression of the major DNA methyltransferases, DNMTs 1, 3a and 3b, and found a significant induction of DNMT3a mRNA expression in FTLD patients compared to controls. DNMTs are essential for proper nervous system development [44,45] and are expressed in the adult brain including postmitotic neurons, where they play a role in synaptic and behavioral plasticity [46]. Overexpressing DNMT3a in LCLs significantly reduced GRN promoter activity and resulted in significantly reduced GRN mRNA expression levels (Figure 6), whereas overexpression of DNMT1 did not have inhibitory effects on GRN promoter activity and GRN mRNA expression (Additional file 1: Figure S4). This finding is in line with the fact that DNMT1 is the maintenance DNA methyltransferase (during mitosis) while DNMT3a and 3b are *de novo* methyltransferases and are thus crucial for adding new methyl groups to CpG dinucleotides [47]. Thus, elevated expression of DNMT3a in the cortices of FTLD patients might add to neuronal vulnerability and cell loss in addition to controlling GRN expression.

Epigenetic therapies using DNMT inhibitors and histone deacetylase (HDAC) inhibitors have been approved by the U.S. Food and Drug Administration (FDA) for cancer chemotherapy [48]. Recent evidence from cell culture and animal studies suggests that HDAC inhibitors, such as sodium butyrate or trichostatin A can improve memory formation and cognition in models of neurodegenerative diseases [49,50]. Thus, correcting epigenetic abnormalities is a promising therapeutic strategy for neurodegenerative diseases. Novel approaches towards treatment of FTLD have attempted to pharmacologically induce GRN protein levels [20,51]. Treatment of cells with the DNA-demethylating drug DAC preferentially reactivates genes silenced by *de novo* methylation. In our study, continued DAC treatment led to a highly significant re-expression of GRN in human lymphoblast cells (Figure 2). Thus, targeting the DNA methylation machinery in FTLD might be a potential strategy to elevate GRN levels and, hence, offer therapeutic options for FTLD patients. Interestingly, infusion of zebularine, a DNA methyltransferase inhibitor, into mouse brain resulted in immediate DNA demethylation of several genes leading to enhanced transcription [52]. In human and murine brain, GRN is predominantly expressed in neurons and microglia [11,53]. We also found a robust increase of GRN expression in the murine microglia cell line BV-2 upon chronic DAC-treatment (Additional file 1: Figure S5), suggesting that DAC-treatment also elevates GRN in brain derived cells. As epigenetic drugs emerge as acknowledged cancer therapeutics [54], treating FTLD and other neurodegenerative disorders with DNA demethylating drugs and/or HDAC inhibitors might be a promising future perspective [55].

## Conclusion

In summary, we have demonstrated that DNA methylation in the GRN promoter region physiologically regulates GRN expression. In brains from FTLD patients, the promoter region of GRN is aberrantly methylated, which may be a novel risk factor for the development of FTLD. Future studies will elucidate whether treatment with DNA demethylating drugs not only restore adequate GRN expression, but also delay or prevent FTLD progression.

## Methods

### Human derived material

The Ethical Committee of the University of Antwerp and the Antwerp University Hospital approved collection of biomaterials of patients and control individuals for clinical, pathological, genetic and functional studies of neurodegenerative brain diseases. EBV-transformed lymphoblast cell lines from 15 neurologically healthy individuals and two FTLD patients were provided by the Flanders-Belgian biobank of the Neurodegenerative Brain Diseases group of the VIB Department of Molecular Genetics (VIB DMG), Antwerp, Belgium. Tissue from frontal cortex of 10 FTLD patients and 5 neurologically healthy control individuals was provided from the Antwerp Brain Bank of the Institute Born-Bunge at the University of Antwerp, Antwerp, Belgium. The FTLD patient group consisted of 4 patients with FTLD-TDP brain pathology with unidentified genetic cause, 4 patients with FTLD-TDP brain pathology due to a GRN mutation [10], and 2 FTLD patients carrying a VCP mutation [32]. Additionally, frontal cortex tissue from 10 sporadic FTLD-TDP patients with unknown genetic cause and 10 neurologically healthy controls were obtained from the MRC London Neurodegenerative Diseases Brain Bank, part of the Brains for Dementia Research network (http://www.brainsfordementiaresearch.org.uk/). The AD patient group consisted of 6 patients with no known genetic cause of disease, one patient carrying a mutation in the gene for Amyloid Precursor Protein (APP 369-C/G) [33], and one patient carrying a familial mutation in the Presenilin1 (PSEN1, P264L) gene [34]. The PD patient group consisted of 8 patients without known genetic cause of disease (Table 1).

For DNA and RNA extraction 30–50 mg of fresh-frozen frontal cortex was ground in liquid nitrogen. DNA was isolated using the DNeasy Blood & Tissue Kit (Qiagen) and total RNA extraction was performed using the Ribopure Kit (Ambion, Applied Biosystems) or using TRIzol® (Invitrogen) and treated with DNase (Turbo DNase Kit; Ambion, Applied Biosystems). The integrity and quality of all human RNAs was verified by Agilent Bioanalyzer 2100 analysis. Due to different post-mortem delays, RNA quality varied between brain samples. According to recent publications, an RNA integrity number of 3.5 was considered as cutoff for subsequent qPCR analyses [56]. Samples with poor melting curve quality were discarded.

### SNP genotyping

The patients and control individuals obtained from the VIB DMG biobank were genotyped for genetic variants in TMEM106b rs1990622 [30,57], GRN rs5848 [31] and SORT1 rs646776 (van der Zee et al. unpublished data). LCLs and brain samples obtained from the MRC London Neurodegenerative Diseases Brain Bank, part of the Brains for Dementia Research network, were genotyped by subjecting DNA to quantitative real time PCR using Taqman probes (Life Technologies, TMEM106b rs1990622, GRN rs5848) according to the manufacturer’s instructions. Genetic variants in SORT1 rs646776 were analyzed by PCR amplification (sense primer: CCAGAAGGCCCCACCGGGA, antisense primer: CCCGTGCAGCCTCTCCCACC) and subsequent sequencing (GATC, Konstanz, Germany). GGGGCC hexanucleotide repeat expansions in C9orf72 were assessed as described in [6,58].

### Cell culture and drug treatment

HEK 293FT and microglia BV-2 cells were maintained in DMEM Glutamax cell culture media (Life Technologies), supplemented with 10% fetal calf serum (Sigma Aldrich) and non-essential amino acids (Life Technologies). Epstein Barr virus transformed lymphoblast cells [12,59] were cultured in RPMI 1640 medium (Life Technologies), supplemented with 10% fetal calf serum (Sigma Aldrich) and glutamine (Life Technologies). 5 × 10^5^ cells per ml were seeded and treated with 0.5 μM (LCLs) or 0.13 μM (BV-2) 5-aza-2′-deoxycytidine (DAC, Sigma Aldrich), dissolved in DMSO. Due to the low half-life of DAC, the cell culture media and DAC was replaced daily. After treatment for 3, 5, 7 and 9 days, supernatant was collected, cells were harvested and further analyzed.

### DNA isolation

DNA was isolated from lymphoblast cell lines using the DNeasy Blood & Tissue Kit (Qiagen) according to the manufacturer’s instructions.

### Quantitative methylation analysis MassARRAY (Sequenom)

DNA methylation at individual CpG units was quantified using the MassARRAY platform, as previously described [22]. Briefly, bisulfite converted DNA (using the EZ DNA methylation kit, Zymo Research) was PCR-amplified (for primer information see Additional file 1: Table S1), *in vitro* transcribed using T7 RNA polymerase, cleaved by RNase A and subjected to matrix-assisted laser desorption/ionization time-of-flight mass spectrometry (Sequenom). Mass shifts of 16 Da introduced by the initial bisulfite conversion led to distinct signal patterns for methylated and non-methylated DNA templates, which allowed for proper quantification of DNA methylation.

### RNA isolation, reverse transcription and quantitative PCR

RNA from lymphoblast and microglia cell lines was isolated with TRIzol® (Life Technologies) followed by DNase digestion (Qiagen). cDNA synthesis was carried out using the Taqman MicroRNA Reverse Transcription Kit (Applied Biosystems) using random hexamer primers (Sigma Aldrich). Quantitative Real Time PCR of human and murine GRN (Hs00173570_m1, Mm01245914_g1, Life Technologies) and PGK1 (Hs00943178_g1, Mm00435617_m1, Life Technologies) was performed using Taqman microRNA mastermix following standard protocols. All other genes were analyzed using the SsoFast Evagreen Supermix (BioRad, for primer information see Additional file 1: Table S2). All samples were run in triplicates and normalized to the housekeeping genes PGK1 and/or GAPDH. Relative mRNA abundance was calculated with the ΔΔC_t_ method.

### GRN ELISA

The supernatant of lymphoblast cell lines was diluted 1:1 with PBS and analyzed by ELISA as described elsewhere [51].

### Isolation of primary rat cortical neurons, transfection and luciferase assay

Primary cortical neurons were prepared from rats as described previously [60]. The GRN promoter region (−2423 to +207 bp relative to transcriptional start site, for primers see Additional file 1: Table S1) was cloned directly into the CpG-free vector pCpGL and *in vitro* methylated using recombinant DNA methyltransferase M.SssI (New England Biolabs). The unmethylated control vector was obtained by omitting S-adenosylmethionine from the M.SssI methylation reaction mix [27]. HEK 293FT cells and primary rat cortical neurons (five days in culture) were transiently transfected in 96-multiwell plates using 0.3 μl (HEK 293FT) or 0.25 μl (primary neurons) Lipofectamine 2000 (Life Technologies), 80 ng of reporter plasmid and 80 ng of *Renilla* luciferase expression vector [60]. After 48 h (HEK 293FT) or 72 h (primary neurons), cells were assayed for firefly and *Renilla* luciferase activity using the dual luciferase reporter assay system (Promega).

### Lentiviral transduction

For lentiviral overexpression of DNMT1 and 3a, the coding sequences of DNMT1 and 3a were cloned into a lentiviral vector under the control of the human ubiquitin C promoter (for primers see Additional file 1: Table S1) [60]. Lymphoblast cells (5 × 10^5^ cells/ml) were transduced and harvested for RNA isolation five days later.

### Statistics

Results are presented as mean ± standard deviation (SD) or standard error (SEM) as indicated. For statistical evaluation one-way ANOVA, Student’s t-test, Mann Whitney U test or Kruskal Wallis test using the GraphPad Prism 5 Software was applied. Values of *p* < 0.05 were considered as statistically significant. Benjamini Hochberg multiple testing was carried out using an estimated false discovery rate (FDR) of q = 0.25.

## Competing interests

The authors declare that they have no competing interest.

## Authors’ contributions

JBS, RC and DE designed the project and wrote the manuscript. JBS performed most experiments. RC and OM performed MassARRAY analyses. KR performed *C9ORF72* genotyping analyses. JvdZ, MC and CVB generated mutation and genotype data of brain samples from selected patients and/or controls, provided lymphoblast cell lines of mutation carriers and extracted RNA of neuropathological diagnosed brain samples of selected patients and/or control individuals, SE and PPDD performed brain autopsy and provided clinical and neuropathological research data. CP and DE supervised research. All authors read and approved the final manuscript.

## Supplementary Material

Additional file 1 Supporting information. Click here for file
