# Supplementary material for: Promoter DNA methylation regulates progranulin expression and is altered in FTLD
Source: Acta Neuropathol Commun. 2013 May 13;1:16. doi: 10.1186/2051-5960-1-16 (PMC3893557; doi:10.1186/2051-5960-1-16)
Supplement: Additional file 1 — Supporting information. [file 2051-5960-1-16-S1.pdf]

Supporting information:

Supplementary Figure 1:

```
taaaaatggtcttaaactgattgtaatgatgctgcataactctcttaagacactaaaaccattgagttgtacacttt
                                   1*                                      2*
aatttttttgtgttggCGggggtggatggagtctCGctctgttgtccaggctggaatgcagtggcaCGatctCGgttt
                   3                                                     4     5
actgcaacctctgcatccCGggttcaagCGattctcctgcctCGgcctcctgagtagggaattacagacctCGttatC

GtggcaccttacccttctgatgttaaaaaaaaaaaaaaaaagagCGagagagagagagagaaacatttgtgaagtagg
                                                                        6*
ttgttgagtctcagcactattgaccttttgggcaggatacttctttgttgtggggattgttctgtgtgtCGtgtgat

gtttagtgggattgctggcccttacctaccagatgccagtgtccctccaccctgagttgtgacaacccagattgtctc
                         7            8*              9
cagacactcctaaatgtccctggcCGgcaaaattgcCGctgctcaagaatcaCGgctttgaCGattagactttgtgat

atttgtttcagtctgtttaggtttttttcttctacctgtatttttttctggttctgggtggttgtaattagtaggtt

attgatCGattcacctaacatttcatgaaagtttcatgtgtgtgtgtgtttcaatagaagcataaactatactcccta

gtctcaagatacacaggaaggaaaataagcacaaatgtgtcaccagggcacagactagtactaggtcctcagcaggcc
             10                                                           11*
aggtgtcttatcCGctgtctgggtctgctctagctccaggcttagaaccccctgccacaCGactccacagctCGgttgg
                                 12
caccctttccctcctcCGacttctgctgcctCGagcttggttagccatccccctgcccctgcctcatcctcagctcca
                                                  13         14
gttccttgctcaggctgcagcagtctccatcccctgtgcagacactgcCGttcctccaCGgcccagtatcaggctttc

cctgggcctctcctctctcctggcccatctcccatcatccatctctgcctggcccaggccctttggcaccaagcaggc

tgactcttgtcactggctaatctgttctgtggtacattttctctcctcaccctcccatatcaattcctCGaaggcagg

gcCGatctggagactaggaagccacttctctttCGacagcccccaccacagcccagccCGtgccaggcacccagcagc

tcctgaagcccactggcattgaacatggcattcaatccctgccaagcctgcccttcccatctggtttcccagggctct

ctcccaacacctcctcctccacctgccagttaaaatcttcccagactcagctcaaggagatgctcctaaggtggaatg

aaatctcttcttccccacctggagacaatctacttcctctccctacacctggcaactggCGcacaaccttgtatctta

aattagattcagcctgagactgtctcccaccaatccctgctccctgtcctgctgagcaccttgaggaaagggctttgg

ggctgtttatctttgtcctggaaaccatccttcaactcactctggggcctgcctagcatgtcaacCGagtttggagaa

tagggcagaatagggcaggacaggacaggacaagacagggcaggataggataggagCGagccagctcagtagctcaca
            DAC1/2         DAC1/2       DAC3                         DAC4
            15/16           15/16        17                           18
tttgtaatcccagCGccttgggggctgCGgtaggagaatCGctttgggagcaggagttgcaggcCGcagtgagctat
           DAC5                                          DAC6/7/8
gatcagcttgggCGactgagCGagaccctgtctctaaaacaaacacacaagtcCGggCGCGgtggctcatgcctgtaa
                  DAC9      DAC10
tcttagcactttgggaggcCGaggtgggCGgatcaCGaggtcaagaaatCGagaccatcctggccaacatggtgaaac
DAC11                                                             DAC12
  19                                                                20
ccCGtctctactaaaaatacaaaaattagctgggCGtggtggtgCGCGcctgtagtcccagctactCGggaggctgag
         DAC13     DAC14                   DAC15
          21        22
gcaggagaatCGcttgaaccCGggaggcagaggttgcagtgagcCGagatCGtgccactgcactccagcctggCGaca
                                              23
gagtgagactcCGtctcagaacaaacaaacaaaaggatagaaaggCGagcacaaatattcccaattcataacactccc
 24                     25/26                                 27
tCGcactgtcaatgccccagacaCGCGctatcatctctagcaaactcccccaggCGcctgcaggatgggttaaggaag
 28                                  29
gCGaCGagcaccagctgccctgctgaggctgtccCGaCGtcacatgattctccaatcacatgatccctagaaatgggg

tgtggGGCGAGAGGAAGCAGGGAGGAGAGTGATTTGAGTAGAAAAGAAACACAGCATTCCAGGCTGGCCCCACCTCTA
```

Promoter region of GRN. All CpG sites are marked in red and all CpG units analyzed by MassARRAY are numbered above. PCR amplicons are highlighted in yellow (A-1), green (A-2), blue (A-3), purple (A-4) and grey (A-5). A-DAC is underlined and CpG units in A-DAC that are analyzed by MassARRAY are numbered (DAC-#). Exon 1 is displayed in capitals and the transcriptional start site is marked with an arrow. In LCLs, DNA methylation at CpG units 1, 2, 6, 8 and 11 was significantly correlated to GRN mRNA expression and net secretion (asterisks).

Supplementary Figure 2:

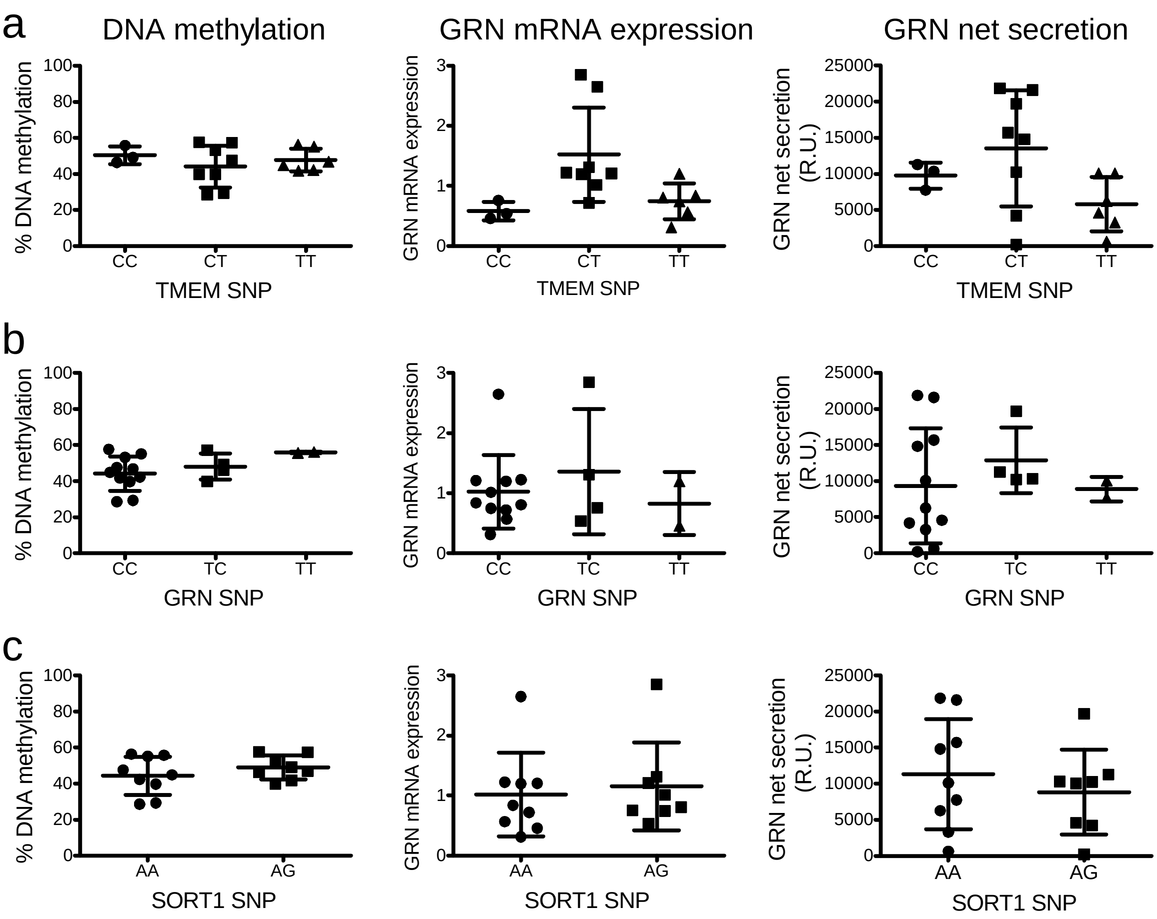

Genetic variations in TMEM106b, GRN and SORT1 do not correlate with DNA methylation levels in amplicon A-1 and GRN mRNA expression/net secretion in LCLs. SNPs for TMEM rs1990622 **(a)**, GRN rs5848 **(b)** and SORT1 rs646776 **(c)** were analyzed by Taqman genotyping assays or PCR amplification with subsequent sequencing. Patients with different genetic variants did not show any difference in GRN promoter methylation in amplicon A-1 (left panel) . GRN mRNA expression levels were quantified by qPCR and normalized to PGK1. GRN mRNA expression levels did not differ in patients with different genetic variants (center panel). GRN net secretion was quantified by ELISA and did not differ in LCLs with different genotypes (right panel). Mean ± SD, ANOVA, Student's t-test.

Supplementary Figure 3:

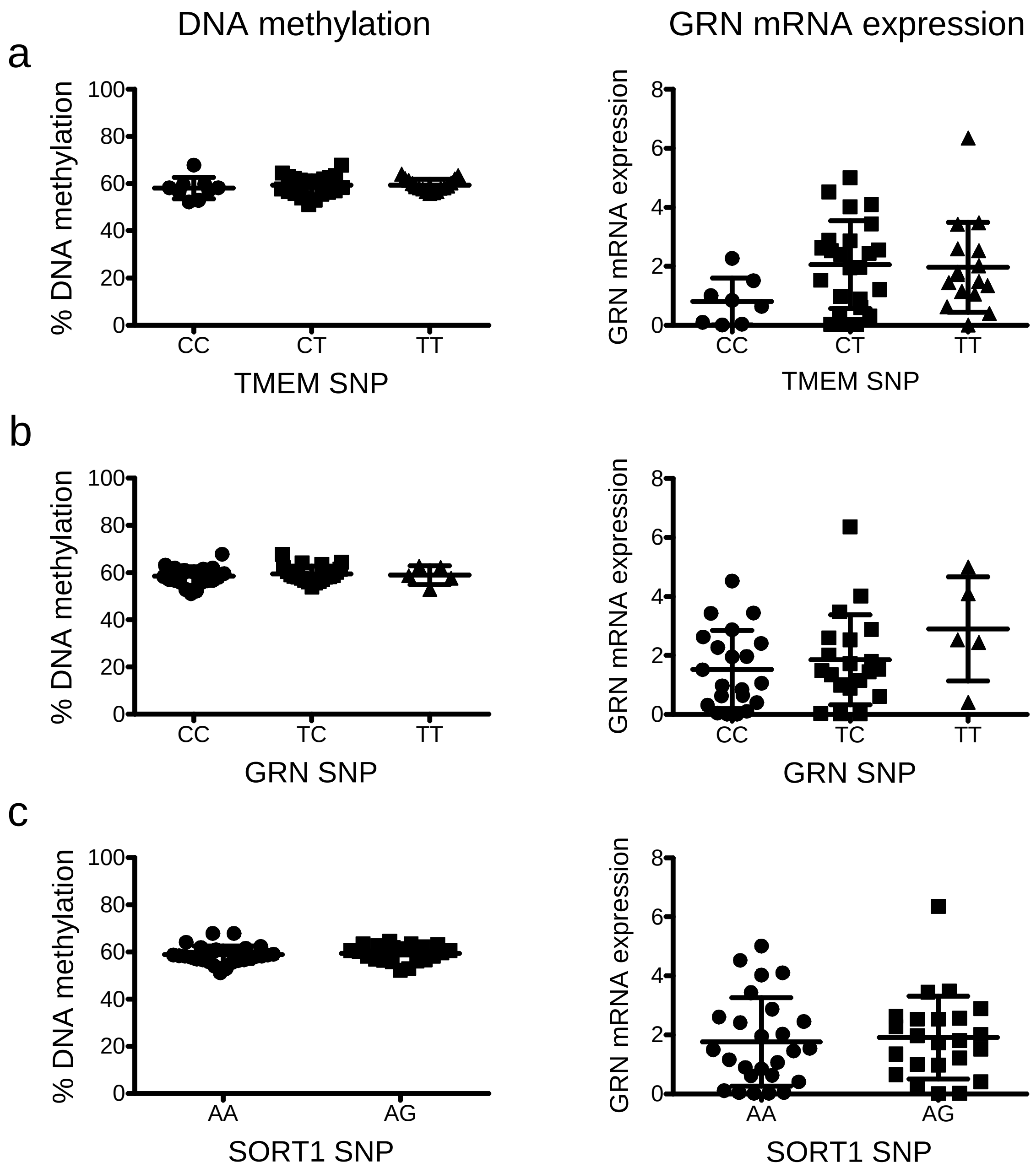

Genetic variations in TMEM106b, GRN and SORT1 do not correlate with DNA methylation levels in amplicon A-1 and GRN mRNA expression in human brain tissue. SNPs for TMEM rs1990622 **(a)**, GRN rs5848 **(b)** and SORT1 rs646776 **(c)** were analyzed by Taqman genotyping assays or PCR amplification with subsequent sequencing. Patients with different genetic variants did not show any difference in GRN promoter methylation in amplicon A-1 (left panel) . GRN mRNA expression levels were quantified by qPCR and normalized to PGK1. GRN mRNA expression levels did not differ in patients with different genetic variants (right panel). Mean ± SD, Wilcoxon rank sum test.

Supplementary Figure 4:

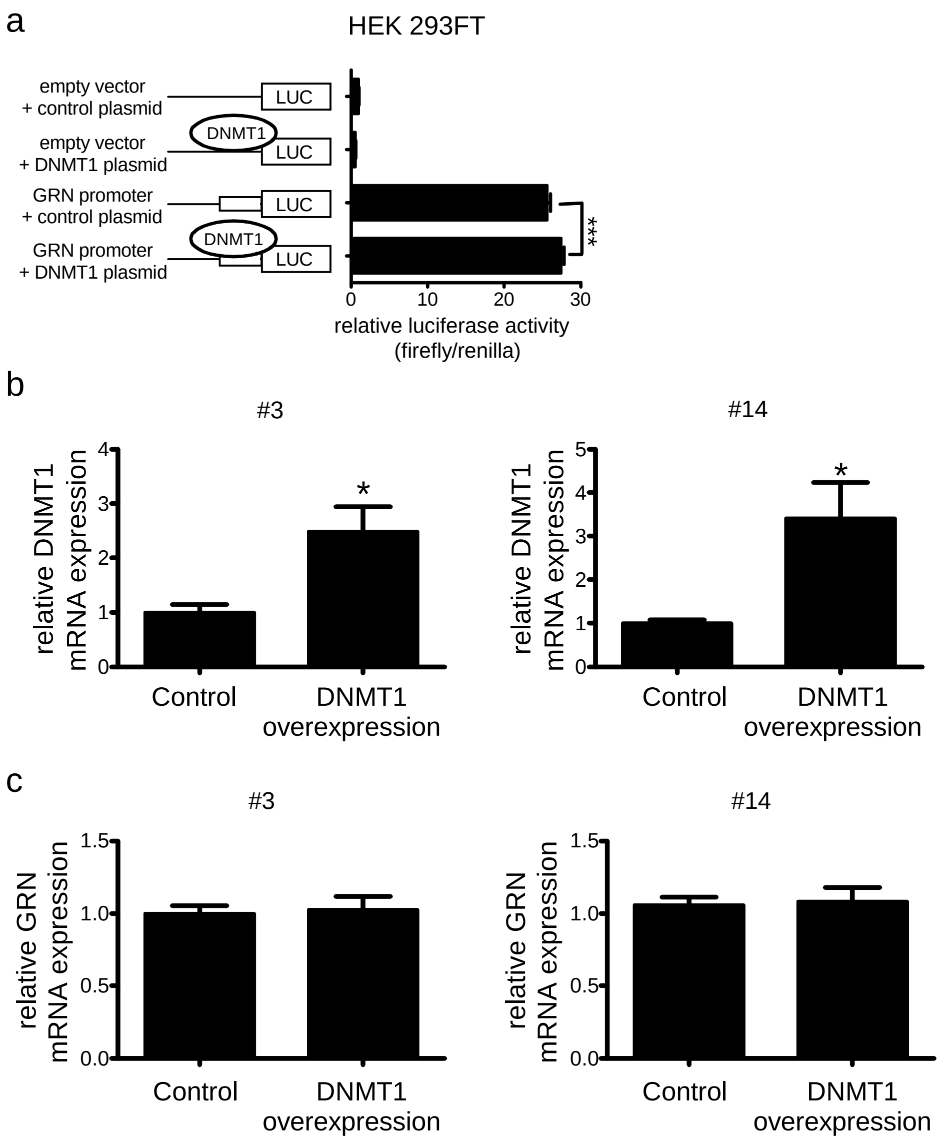

Overexpression of DNMT1 does not alter GRN promoter activity and does not reduce GRN mRNA expression in LCLs

(a) pCpGL plasmid containing the GRN core promoter and a DNMT1 overexpression construct were transiently transfected in HEK 293FT cells. Relative luciferase activity was determined by normalizing firefly luciferase against *Renilla* luciferase activity. Empty vectors were used as negative control. Firefly luciferase expression was slightly increased by DNMT1 overexperssion. Mean ± SEM, n ≥ 3. *** p<0.001, ANOVA with Tukey's Multiple Comparison test. (**b**) Lentiviral expression of DNMT1 in LCLs #3 and #14. Overexpression was verified by qPCR five days after viral transduction. n=4, mean ± SEM, * p<0.05, Student's t-test. **(c)** GRN mRNA expression levels were unchanged in DNMT1 overexpressing LCLs as quantified by qPCR and normalized to PGK1 expression levels. n=4, mean ± SEM, * p<0.05, Student's t-test.

Supplementary Figure 5:

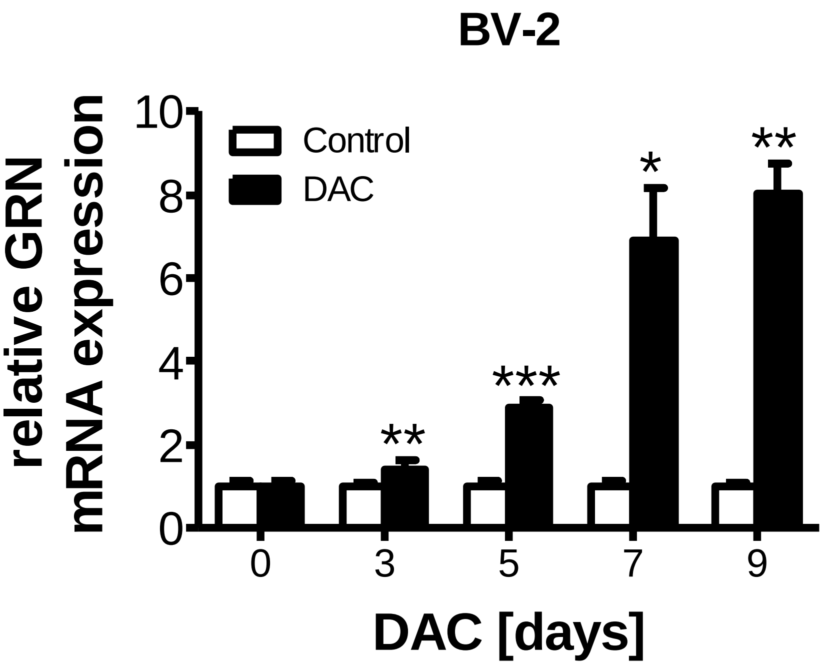

Treatment with the DNMT-inhibitor 5-aza-2'-deoxycytidine (DAC) significantly increases GRN mRNA and protein expression in the murine microglia cell line BV-2. Treatment of the murine microglia cell line BV-2 with 0.13 µM DAC for 9 days significantly increased GRN mRNA expression, while GRN expression in control treated cells did not change. GRN mRNA expression levels were quantified by qPCR and normalized to PGK1. Mean ± SD, n=3, * $p<0.05$, ** $p<0.01$, *** $p<0.001$, Student's t-test.

Supplementary Table 1: Primers used for MassARRAY and cloning

Primer (5'-3')	Forward	Reverse
A-1	aggaagagagtttttgtggtgataaaaatggttt	cagtaatacgactcactatagggagaaggctcacaactcaaaataaaaaaacactaaca
A-2	aggaagagaggtttagtgggattgttggttttta	cagtaatacgactcactatagggagaaggctttaaccaataacaaaaatcaacctac
A-3	aggaagagagattttggggtttgtttagtatgttaat	cagtaatacgactcactatagggagaaggctcccaaactaatcataactcactac
A-4	aggaagagagagattattttggttaatatggtgaa	cagtaatacgactcactatagggagaaggctactcactacaacctctacctccc
A-5	aggaagagagttagaataaataaataaaaggataga	cagtaatacgactcactatagggagaaggctcacaccccatttctaaaaatcatat
A-DAC	aggaagagaggttagtttagtagtttatatttgtaat	cagtaatacgactcactatagggagaaggcttctatccttttatttatttattctaa
GRN promoter	aggtagaaagtagatcagtggttg	tccgactccgcggtccttgggca
GRN promoter short	gcagctcctgaagcccactggcattg	tccgactccgcggtccttgggca
DNMT1	aaaggcgcgcctatgccggcgcgtaccgccccagcccg	agagaattcctagtccttagcagcttcctcctcct
DNMT3a	agaggcgcgcctatgaatgctgtggaggaaagccaggcctc	catgaattcccatgtcccttacacacaagcaaaatattccttc

Supplementary Table 2: Primers used for qPCR

Primer (5'-3')	Forward	Reverse
DNMT1	cccctgagccctaccgaat	ctcgctggagtggacttgtg
DNMT3a	aagcagggcaaagaccagca	agcggagcgaagaggtggcg
DNMT3b	gacttggtgattggcggaa	ggccctgtgagcagcaga
GAPDH	ctgcaccaccaactgcttag	gtcttctgggtggcagtgat

Supplementary Table 3: Linear correlation of DNA methylation at individual CpG units and GRN secretion in lymphoblast cell lines. R^2 and p-value are given. Sites that were still significant after Benjamini Hochberg multiple testing and FDR correction (estimated q=0.25) are highlighted in bold.

		GRN mRNA expression		GRN secretion	
**Amplicon**		R^2	P value	R^2	P value
A-1	**1**	**0.4959**	**0.0072**	0.3255	0.0417
	**2**	0.2568	0.0379	**0.3792**	**0.0085**
	3	0.09604	0.2261	0.3024	0.0222
	4	0.08346	0.2778	0.001575	0.884
	5	0.1689	0.1013	0.1495	0.1253
	**6**	**0.2796**	**0.0291**	0.2777	0.0297
A-2	7	0.000561	0.9281	0.02426	0.5505
	**8**	**0.2932**	**0.0248**	0.04152	0.4328
	9	0.0001	0.9696	0.002471	0.8497
	10	0.003158	0.8304	0.1883	0.0818
	**11**	**0.3071**	**0.021**	0.1462	0.1298
	12	0.03725	0.5479	0.02275	0.6399
	13	0.001864	0.8693	0.004069	0.8078
	14	0.1961	0.0751	0.02055	0.5831
A-3	15/16	0.04838	0.3963	0.001432	0.8853
	17	0.02059	0.5827	0.008807	0.7201
	18	0.000344	0.9437	0.0359	0.4664
A-4	19	0.1922	0.0784	0.2195	0.0579
	20	0.1465	0.1295	0.1038	0.2074
	21	0.2534	0.0394	0.2435	0.0441
	22	0.1465	0.1295	0.1038	0.2074
A-5	23	0.02751	0.6065	0.003534	0.8544
	24	0.006835	0.7524	0.09132	0.2384
	25/26	0.1442	0.1328	0.009952	0.7032
	27	0.00185	0.8698	0.001644	0.8772
	28	0.02751	0.6065	0.003534	0.8544
	29	0.1313	0.153	0.008449	0.7257
